# Exogenous Volatile Organic Compound (EVOC^®^) Breath Testing Maximizes Classification Performance for Subjects with Cirrhosis and Reveals Signs of Portal Hypertension

**DOI:** 10.3390/biomedicines11112957

**Published:** 2023-11-01

**Authors:** Giuseppe Ferrandino, Federico Ricciardi, Antonio Murgia, Iris Banda, Menisha Manhota, Yusuf Ahmed, Kelly Sweeney, Louise Nicholson-Scott, Lucinda McConville, Olga Gandelman, Max Allsworth, Billy Boyle, Agnieszka Smolinska, Carmen A. Ginesta Frings, Jorge Contreras, Claudia Asenjo-Lobos, Viviana Barrientos, Nataly Clavo, Angela Novoa, Amy Riviotta, Melissa Jerez, Luis Méndez

**Affiliations:** 1 Owlstone Medical, 183 Cambridge Science Park, Milton Road, Cambridge CB4 0GJ, UK; 2 Department of Pharmacology and Toxicology, School for Nutrition and Translational Research in Metabolism (NUTRIM), Maastricht University Medical Center, 6229 HX Maastricht, The Netherlands; 3 Unidad de Gastroenterología y Endoscopía, Clínica Alemana, Facultad de Medicina Clínica Alemana, Universidad de Desarrollo, Santiago 7650568, Chile; 4 Unidad de Endoscopia, Hospital Padre Hurtado, Santiago 8880465, Chile; 5 Centro de Estudios Clínicos, Instituto de Ciencias e Innovación en Medicina (ICIM), Facultad de Medicina Clínica Alemana, Universidad del Desarrollo, Santiago 7610315, Chile; 6 Laboratorio de Fisiología Digestiva, Clínica Alemana, Santiago 7650568, Chile; 7 Nursing School, Universidad de Las Américas, Santiago 8242125, Chile

**Keywords:** breath biopsy, volatile organic compounds, functional diagnostics, non-invasive, MELD

## Abstract

**Background:** Cirrhosis detection in primary care relies on low-performing biomarkers. Consequently, up to 75% of subjects with cirrhosis receive their first diagnosis with decompensation when causal treatments are less effective at preserving liver function. We investigated an unprecedented approach to cirrhosis detection based on dynamic breath testing. **Methods:** We enrolled 29 subjects with cirrhosis (Child–Pugh A and B), and 29 controls. All subjects fasted overnight. Breath samples were taken using Breath Biopsy^®^ before and at different time points after the administration of 100 mg limonene. Absolute limonene breath levels were measured using gas chromatography–mass spectrometry. **Results:** All subjects showed a >100-fold limonene spike in breath after administration compared to baseline. Limonene breath kinetics showed first-order decay in >90% of the participants, with higher bioavailability in the cirrhosis group. At the Youden index, baseline limonene levels showed classification performance with an area under the roc curve (AUROC) of 0.83 ± 0.012, sensitivity of 0.66 ± 0.09, and specificity of 0.83 ± 0.07. The best performing timepoint post-administration was 60 min, with an AUROC of 0.91, sensitivity of 0.83 ± 0.07, and specificity of 0.9 ± 0.06. In the cirrhosis group, limonene bioavailability showed a correlation with MELD and fibrosis indicators, and was associated with signs of portal hypertension. **Conclusions:** Dynamic limonene breath testing enhances diagnostic performance for cirrhosis compared to static testing. The correlation with disease severity suggests potential for monitoring therapeutic interventions. Given the non-invasive nature of breath collection, a dynamic limonene breath test could be implemented in primary care.

## 1. Introduction

Cirrhosis is an end-stage liver disease resulting from long-term exposure to chronic liver injuries of different aetiologies [1]. Globally, the main causes of cirrhosis are hepatitis B and C, alcoholic-related liver disease, and non-alcoholic steatohepatitis (NASH) [2]. Disease progression is often asymptomatic, with up to 75% of the cases diagnosed with manifestation of decompensation defined by hepatic encephalopathy, jaundice, variceal bleeding, or ascites [1,3,4]. Patients with a diagnosis of cirrhosis, before decompensation, can benefit from causal treatments of the underlying aetiology (e.g., viral suppression/eradication, alcohol abstinence, lifestyle improvements) [5], and achieve a life expectancy similar to that of the general population [2]. Conversely, patients diagnosed with decompensation, especially with deadly complications, have a poor prognosis, and can benefit from costly and invasive treatments (i.e., fluid resuscitation, vasopressors, endoscopic band ligation, injection sclerotherapy, transjugular intrahepatic portosystemic shunt (TIPS), paracentesis, lactulose, liver transplant) [5]. The incidence of cirrhosis-related deaths increased from 1.9% of all deaths in 1990 to 2.7% in 2017 [2]. Calls for action [6,7,8] and a statement on the health policy of the European Union [9] emphasised the need for earlier diagnosis to lower the burden of chronic liver diseases and associated mortality.

The detection of cirrhosis in primary care relies on biomarkers with poor specificity and sensitivity [10], while better-performing algorithms rely on tests available in secondary and tertiary care [11,12]. The consequent short-circuit in the diagnostic pathway results in patients who should be referred for treatment but remain undetected until overt decompensation. Overcoming this impasse requires the implementation of novel, more practical, and less invasive diagnostic methods [1]. The analysis of volatile organic compounds (VOCs) in exhaled breath represents an emerging diagnostic means with the potential to develop non-invasive tests for early disease detection in primary care, or at-home testing [13]. The majority of exhaled VOCs stem from blood–air alveolar exchange, and their breath concentration is proportional to the blood concentration and dictated by well-defined physical chemistry properties [14]. A subset of VOCs is mainly introduced with the diet and defined as exogenous volatile organic compounds (EVOCs) [15]. Most of these EVOCs undergo hepatic phase I/II metabolism for excretion in the urine [16,17], with a small fraction of the dose achieving the systemic circulation and excreted unchanged in the breath [18]. Chronic liver diseases, with the associated metabolic and anatomical hepatic alterations, change the excretion route [19] or the systemic bioavailability of certain compounds [20,21,22]. These changes could be used for diagnostic purposes.

Limonene is an EVOC that is ingested mainly through the diet [23]. It is a monoterpene present in citrus fruits and is accepted as a safe product and used in the pharmaceutical and food industry. It is rapidly absorbed in the gastrointestinal (GI) tract [24], where it is distributed to the body, especially in the richly perfused organs, and mainly accumulates in the liver [25], with a small fraction excreted unchanged in the breath after acute exposure [15,18]. Previous studies found elevated breath limonene in subjects with cirrhosis [23,26,27,28,29,30], suggesting that reduced liver function increases the amount of the compound excreted in the lungs, providing a classification performance for a potential limonene breath test. We showed that, in subjects with cirrhosis, levels of breath limonene correlate with biomarkers used as a proxy for hepatic clearance and protein synthesis capacity, but not with biomarkers of liver damage [23]. Consistently, breath limonene was higher in subjects with more advanced cirrhosis, measured as Child–Pugh class [23]. In addition, we found that breath limonene levels also depend on the extent of the dietary exposure [23], which represents a confounder to control in order to maximize the classification performance of a potential limonene breath test [18].

For this purpose, we established an unprecedented approach, in which subjects with cirrhosis or controls ingest a defined dose of limonene in a formulation with high bioavailability, and breath is collected before and at different timepoints after administration.

## 2. Materials and Methods

### 2.1. Study Design and Subjects

This prospective diagnostic accuracy study was designed according to the STAndards for the Reporting of Diagnostic accuracy studies (STARD) guidelines and approved by “Comité Ético Científico de la Facultad de Medicina—Clínica Alemana Universidad del Desarrollo de Santiago, Chile”. All participants provided written informed consent. All procedures were conducted in compliance with the applicable guidelines for the ethical conduct of the study, with origins in the Declaration of Helsinki [31] and Istanbul. The presence or absence of cirrhosis and signs of portal hypertension were established using ultrasound and blood tests according to EASL and AASLD guidelines [32,33]. Participants older than 18 years and weighing more than 60 kg were randomly enrolled between January 2022 and December 2022. Cases of cirrhosis were patients under outpatient control with a follow-up in gastroenterology at two centres in Santiago de Chile. Patients with fever, signs of acute decompensation, or recent hospitalization were excluded. Healthy relatives of the patients and other volunteers were invited to participate. To confirm the absence of liver disease, abdominal ultrasound, and liver laboratory tests (transaminases, bilirubin, and prothrombin time) dated no more than 6 months prior to breath testing were requested. All participants were instructed before the experiment to fast for ≥10 h, to not consume citrus fruits and alcohol the previous day, and to not brush their teeth or use mouthwash in the previous 2 h. Participants provided a first breath sample followed by limonene ingestion (100 mg) in liquid form using an oral fluid medicine syringe (BD Discardit II 309050 BD, Vaud, Switzerland), followed by 200 mL of water to wash potential residual compound from the mouth. Subsequently, post-administration breath samples were collected at scheduled timepoints. Sample size estimation was performed using G*power 3.1 (UCLA, Los Angeles, CA, USA) [34] based on previous findings [23,35]. With the expected effect size (Cohen’s d), and assuming a non-parametric distribution, a sample size of 54 subjects (27 control, 27 cirrhosis) was predicted to provide a power > 0.9. Accounting for a potential 10% sample loss, we aimed to enroll a total of 60 subjects (30 control, 30 cirrhosis).

Ultrasound analysis revealed that one subject enrolled in the control group had undiagnosed liver disease, while a subject with cirrhosis, due to autoimmune hepatitis diagnosed 3 years before breath analysis, showed cirrhosis resolution in a follow-up check. These subjects were initially excluded and treated as a case report.

### 2.2. Breath Biopsy Collection

The acquisition of Breath Biopsy samples was achieved using the ReCIVA^®^ Breath Sampler (Owlstone Medical, Cambridge, UK) [23]. Detailed methods are available in the Supplementary File S1.

### 2.3. Limonene Measurements

Breath samples were analyzed using Breath Biopsy OMNI global VOC analysis [36]. Detailed methods are available in the Supplementary File S1.

### 2.4. Data Handling and Statistical Analysis

Data were analysed using Python (Python Software Foundation, Fredericksburg, VA, USA, V. 3.11) [37] and RStudio (Posit, PBC, Boston, MA, USA, V. 2023.06.1) [38]. Data visualisation was performed using libraries matplotlib (V. 3.7) [39], seaborn (V. 0.12.2) [40], Corrplot (V. 0.92) [41], and ggplot2 (V. 3.4.4) [42].

Limonene exhalation kinetics were analysed using non-compartmental analysis. The PKA NCA package (V. 0.10.2, https://CRAN.R-project.org/package=PKNCA, accessed on 15 March 2023) [43] was used to calculate area under the curve (AUC). Time to peak concentration (Tmax), highest concentration (Cmax), initial concentration (T0), and slope (rate of elimination) parameters were calculated for each subject and summarized by disease group.

Statistical significance between the group medians was tested using non-parametric Mann–Whitney U tests.

Log transformation was performed to bring variables closer to a symmetric distribution.

At each timepoint, the difference in limonene levels between the two groups was evaluated using generalised linear models. Additionally, classification models were built using logistic regression with 5-fold cross validation on an 80/20% training/test split. Performance metric estimates (i.e., AUC, sensitivity, and specificity) obtained from the test set were then averaged to provide a more robust evaluation of the model accuracy. The impact of age on Limonene values was evaluated using a Linear Mixed-Effect’s model to account for the longitudinal nature of the washout data.

The relationship between FIB4, APRI, MELD, and breath limonene in cirrhotic patients was assessed using canonical correlation analysis (CCA) [44]. CCA finds the relationship between two sets of variables measured for the same set of samples and is considered an extension of bivariate correlations. The resulting CCA score plot was generated using the statistically significant canonical variates. The contribution of the original variables to the correlation between the two blocks can be estimated by calculating the canonical loadings, which express the correlation between the original variable and the canonical variate.

## 3. Results

### 3.1. Subjects Characteristics

Breath samples were collected from 29 controls (M: age 43, IQR 38–57 years, m/f, 11/18 (38/62%) and 29 patients with cirrhosis (M: age 59, IQR 54–67 years, m/f, 9/20 (31/69%). Liver condition was confirmed by ultrasound. Study subject details are provided in Table 1. A significant difference in age was observed between study groups (*p* < 0.001. No significant differences were observed for morphometric parameters, except waist circumference (*p* = 0.036).

### 3.2. Limonene Exhalation Kinetic

**Figure 1:**
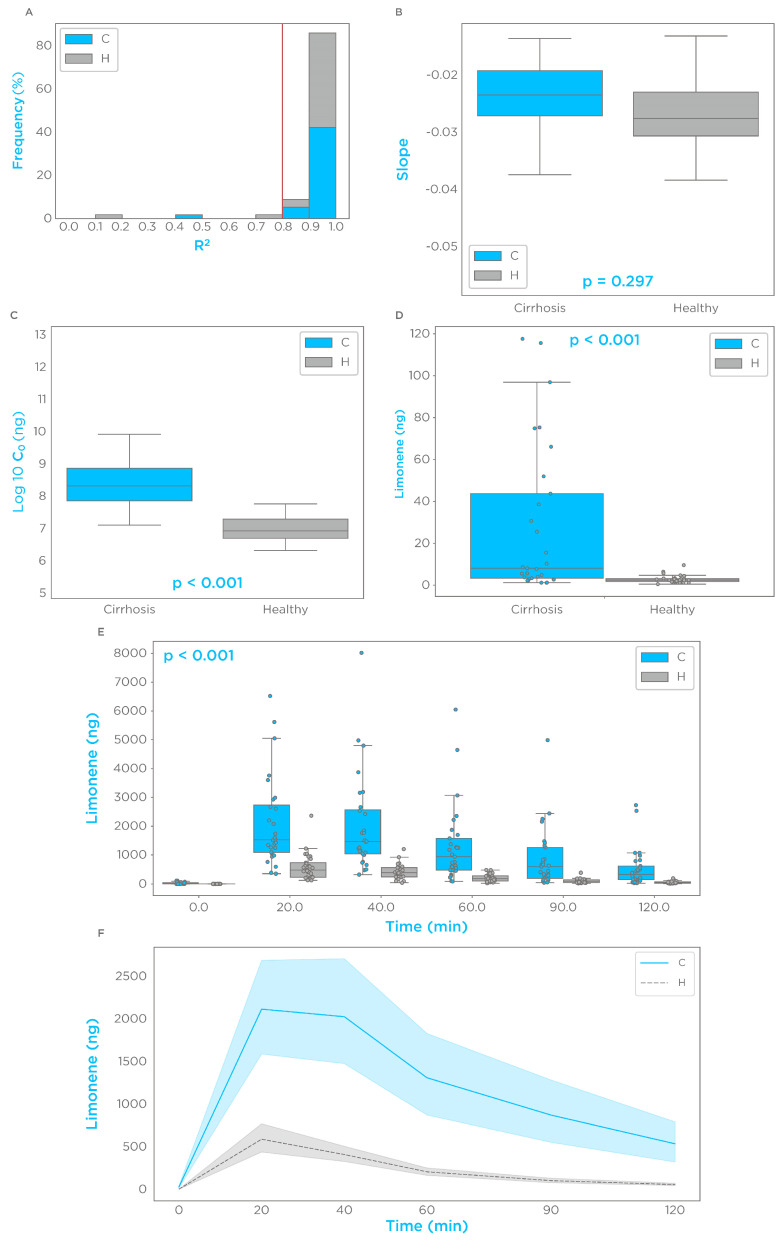
Limonene exhalation shows first-order kinetics and increased bioavailability in subjects with cirrhosis. From semi-logarithmic plots of breath limonene levels as a function of time from T_max_: (**A**) the distribution of R^2^ for all the subjects; (**B**,**C**) boxplots by group of the coefficients of the linear regression representing, respectively, the slope (decay), and the intercept (C_0_); (**D**) enlarged boxplot for breath limonene levels before administration; (**E**) boxplots of breath limonene levels before and after administration at the indicated timepoints; (**F**) mean and 95% confidence interval, by group, of breath limonene levels across the measured timepoints to highlight alterations in systemic bioavailability induced by cirrhosis.

After overnight fasting (ON), at baseline, we detected a GC-MS spectral peak of limonene in all the tested subjects. However, six controls with a low, non-quantifiable peak area were approximated to the limit of quantification (1.18 ng). The ingestion of 100 mg limonene induced a spike in breath of >100-fold the baseline levels in all subjects. More than 90% of the subjects showed a limonene maximal breath amount (C_max_) within 20 and 40 min (T_max_). The investigated timecourse in a semi-logarithmic presentation showed single-phase exponential decay of breath limonene with first-order kinetics (R^2^ > 0.8) in >90% of the subjects (Figure 1A). Limonene breath kinetics for subjects with an R^2^ lower than 0.8 are shown in Supplementary Figure S1. A groupwise comparison of this subset showed no significant differences in the slope at the semi-logarithmic scale (*p* = 0.297) (Figure 1B), while subjects with cirrhosis had a significantly higher Y intercept (C_0_) (*p* < 0.001) (Figure 1C). Groupwise comparisons of all the participants showed that subjects with cirrhosis had higher levels of limonene on breath for each tested timepoint (*p* < 0.001) (Figure 1D,E), with the cirrhosis group presenting with a higher C_max_ and bioavailability (Figure 1F). Limonene breath kinetic data are summarized in Table 2. These data indicate that the effect of cirrhosis on limonene exhalation kinetics mirrors the pharmacokinetic (PK) alterations induced by cirrhosis on drugs with high hepatic extraction, also known as flow-limited [21,22].

### 3.3. Limonene Association with Signs of Portal Hypertension

**Figure 2:**
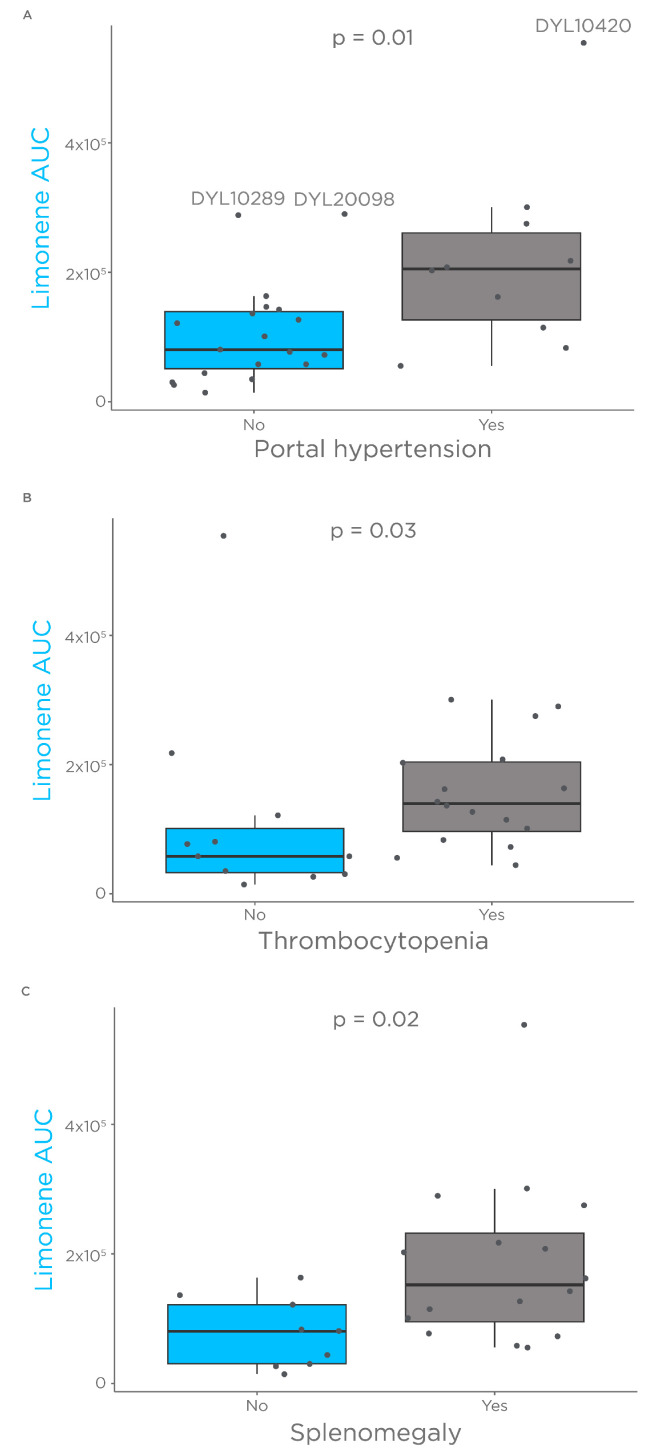
Limonene association with signs of portal hypertension. Comparison of limonene bioavailability estimated as AUC in patients with and without portal hypertension. (**A**) Boxplot of limonene AUC by presence or absence of portal hypertension. (**B**) Boxplot of limonene AUC by presence or absence of thrombocytopenia. (**C**) Boxplot of limonene AUC by presence or absence of Splenomegaly.

Portal hypertension stems from increased intrahepatic vascular resistance [45] and is the main complication that affects the prognosis and quality of life of patients with cirrhosis [46]. As limonene showed an exhalation kinetic similar to the PK of flow limited drugs, we hypothesized that alterations in limonene bioavailability are associated with signs of portal hypertension identified with ultrasound. Consistent with our hypothesis, patients with portal hypertension showed significantly higher levels of limonene bioavailability compared to patients reported without portal hypertension (Figure 2A).

Similarly, patients with thrombocytopenia (platelets count < 150 × 10^9^/L) and splenomegaly (Spleen length > 12 cm) showed increased limonene bioavailability (Figure 2B,C). Interestingly, one subject (DYL10420) with outstanding limonene bioavailability (Figure 2A) was of Child–Pugh class B with the presence of mild ascites. Of the two patients reported not to have portal hypertension (DYL20098, and DYL10289) who showed higher levels of limonene bioavailability (Figure 2A), both were Child–Pugh class A, and one of them was affected by thrombocytopenia. These findings suggest that a limonene breath test could be used to identify cirrhosis subjects who have developed portal hypertension.

### 3.4. Limonene Classification Performance

**Figure 3:**
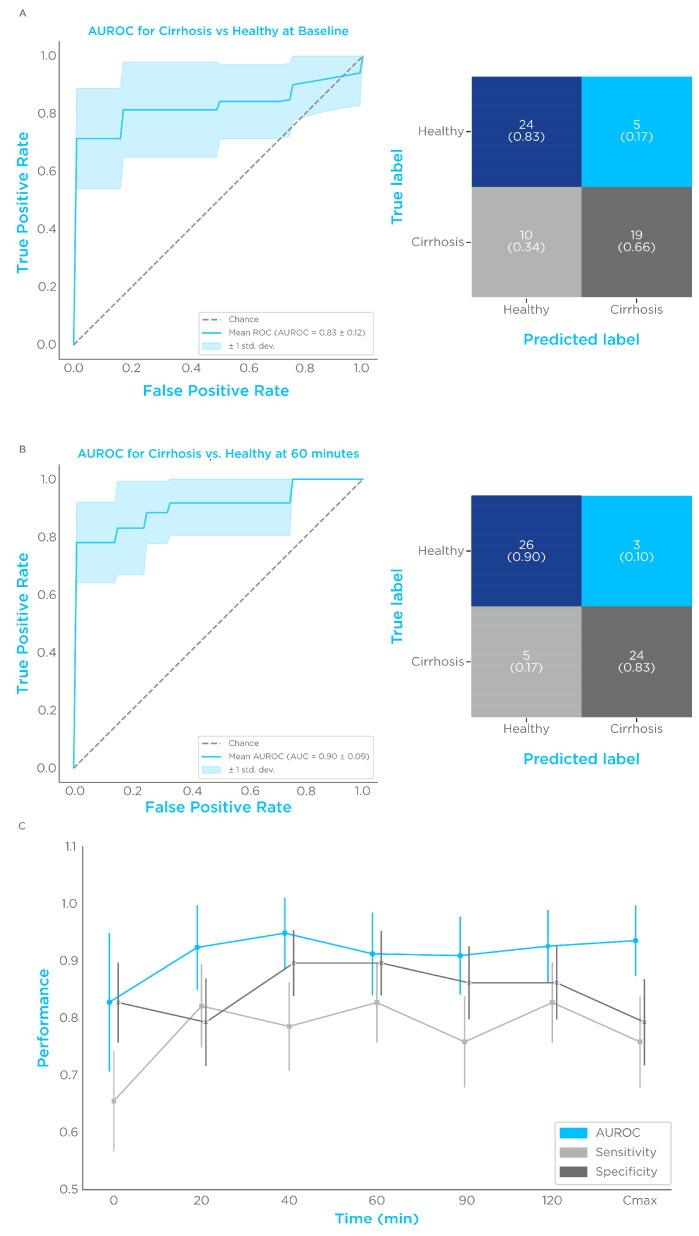
Limonene classification performances. Comparison of breath limonene levels of control vs. cirrhosis. (**A**) ROC plot and confusion matrix before limonene administration, (**B**) and 60 min after administration. (**C**) Visualization of AUROC, sensitivity, and specificity for each timepoint and C_max_. Sensitivity and specificity were measured at the Youden index of the logistic regression function.

Elevated levels of limonene in subjects with cirrhosis were found in several studies [23,26,27,28,29,30,47,48] and were cleared after liver transplant [27], indicating that breath alterations are a consequence of hepatic dysfunction [23,27]. Consistently, after ON fasting we found elevated levels of limonene in the breath of subjects with cirrhosis, which provided a discriminatory performance with an area under the ROC curve (AUROC) of 0.83 ± 0.12 (Figure 3A). The Youden index threshold of the logistic function produced a specificity of 0.83 ± 0.07 and a sensitivity of 0.66 ± 0.09 (Figure 3A). In agreement with the hypothesis that random dietary limonene exposure represents a confounding factor in enhancing classification performance [18], 60 min post-ingestion was one of the best-performing timepoints, with an AUROC of 0.91 ± 0.07, a specificity of 0.9 ± 0.06, and a sensitivity of 0.83 ± 0.07 at Youden index (Figure 3B). Breath washouts for misclassified subjects in relation to the groupwise confidence intervals are presented in Supplementary Figure S2. All the post-administration timepoints showed an improved classification performance compared to baseline (Figure 3C and Table 3). The results of the linear mixed-effect’s model confirmed that age is not a confounder for limonene exhalation kinetics (*p* = 0.623). These findings suggest that a dynamic limonene breath test is a potential high-performing diagnostic test for cirrhosis.

### 3.5. Correlation of Limonene Breath Test with Severity of Cirrhosis and Potential Use as a Prognostic Tool

**Figure 4:**
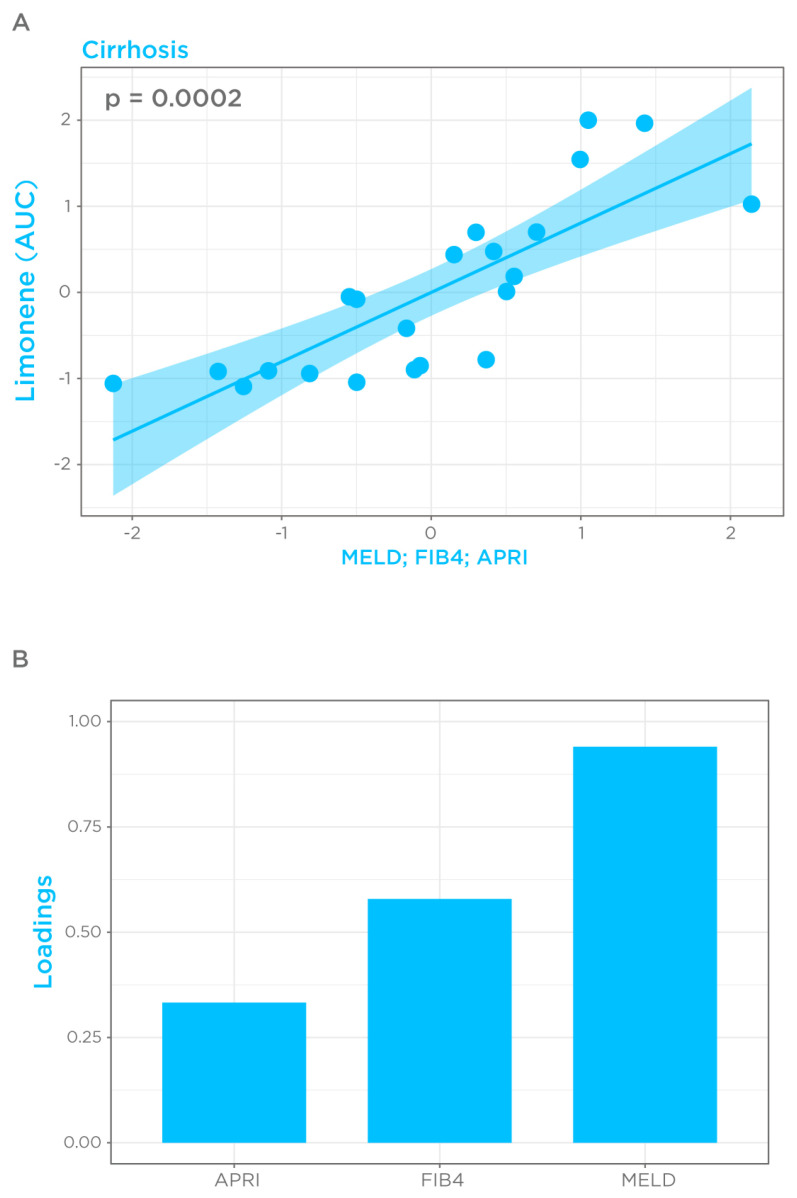
Correlation of limonene bioavailability with MELD, FIB4, and APRI in the cirrhosis group. (**A**) CCA score plot using the first canonical variates of limonene bioavailability, estimated as AUC, and scoring parameters. Each projected data point represents the combined information of limonene AUC and scoring systems of one cirrhotic patient. (**B**) Canonical loadings of the scoring systems representing their correlation between the original variable and its canonical variate and expressing the contribution of each variable to the overall correlation. A total of 6 subjects with incomplete metadata were excluded from analysis.

Given that cirrhosis-induced alterations in limonene kinetics mimic those observed in the PK of flow-limited drugs [21], we considered breath limonene AUC as a proxy of bioavailability in the cirrhosis group, and correlated this parameter with the MELD score for disease severity, and FIB4 and APRI for risk of advanced fibrosis, using the CCA. In the cirrhosis group, breath limonene AUC showed a significant collective correlation with the explored scoring systems on the first dimension (*p* = 0.0002) (Figure 4A). As expected, no significant collective correlation was observed in the control group (*p* = 0.6). MELD showed a higher loading than FIB4 and APRI, of 0.94, 0.57, and 0.33, respectively, indicating that MELD is the main parameter contributing to the correlation (Figure 4B). Correlations between single variables are summarized in Supplementary Figure S3. These data highlight the potential of a limonene breath test to monitor disease progression/regression after therapeutic intervention.

### 3.6. Case Reports

**Figure 5:**
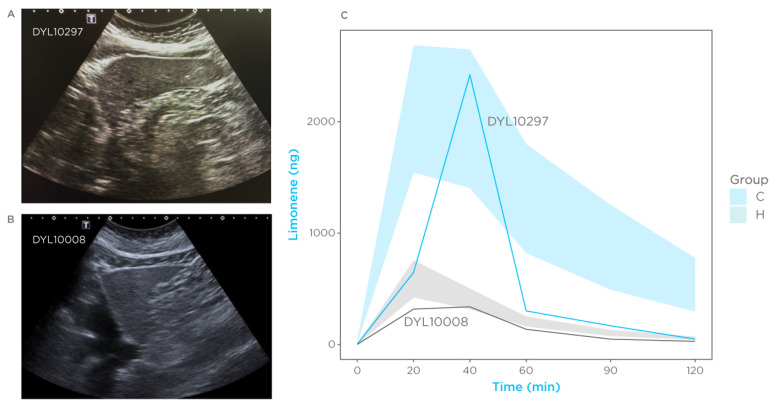
Case reports. Subjects found to have been allocated in the wrong group after ultrasound confirmation of liver condition. (**A**) Ultrasound image of a subject who received a diagnosis of liver disease by enrolling in this study, initially allocated as healthy. (**B**) Ultrasound image of a subject diagnosed with cirrhosis 3 years before the breath tests, who showed recovery after treatment. (**C**) Breath profile of these subjects. Shaded areas represent the 95% confidence interval for the cirrhosis group (blue) and control (grey) for comparison. Each line represents a subject.

The abdomen ultrasound of one subject (ID: DYL10297) enrolled as a control showed a normal liver size with cholecystectomy. However, the parenchyma was slightly heterogenous. Increased parenchymal echogenicity was compatible with steatosis. Focal lesions were not observed. The bile duct was 9 mm at the hepatic hilum, with no obstructions (Figure 5A). Mild chronic liver disease was suspected, and a clinic pathological correlation was suggested. Remarkably, the exhalation kinetic of this subject was similar to that observed in cirrhosis patients (Figure 5C), with a C_max_ at 40 min of 2421 ng. Consistently, this patient was allocated as cirrhotic by the classification model.

Another subject (ID: DYL10008) was diagnosed with cirrhosis due to autoimmune hepatitis three years before the limonene breath test. This subject was initially enrolled in the cirrhosis group. However, a follow-up confirmatory ultrasound showed a liver of normal size with mild steatosis, a 15 mm pseudo-nodular hyper-congenic focus in segment IV with no evident vascularization, and a regression of cirrhosis following immunosuppressant treatment (Figure 5B).

The limonene breath profile of this subject is shown in Figure 5C and resembles that of the healthy group. These cases support the utility of a limonene breath test for diagnostic and prognostic purposes.

## 4. Discussion

Establishing the diagnosis of cirrhosis before symptomatic complications is very important to commence etiological treatments and preserve liver function. An accurate, non-invasive, and simple-to-apply test could be of great clinical utility.

Here, we demonstrated for the first time that dynamic limonene breath analysis provides an excellent diagnostic performance for cirrhosis, with high sensitivity and specificity. Given the low invasiveness and simplicity of breath collection, a diagnostic test relying on this approach could complement or even replace the current methods used in primary care.

Breath analysis has been investigated for the detection of several diseases [13,49,50]. However, especially for liver diseases, potential biomarkers are mainly of exogenous origin [18]. Consequently, their breath levels depend on dietary habits [23], and subjects with cirrhosis whose clearance remains higher than exposure would be classified as a false negative by a static test, whereas exposure to a standard dose before breath collection overcomes this limitation. We found that alterations in limonene breath levels at baseline, before administration, align with previous reports [27], although we used an earlier-stage cirrhosis cohort with the majority of the subjects having Child–Pugh class A in the lower range of the MELD score. As levels of limonene in breath reflect those of the venous blood [14], we compared the dynamic portion of our study with previous studies exploring limonene PK and alterations in cirrhosis [22,51]. Limonene appears in the circulation rapidly after oral administration, with a distribution volume that is greater than total body water, indicating extensive tissue binding and distribution [24,52]. Oral doses primarily accumulate in the liver, with a minor fraction reaching the systemic circulation and richly perfused organs [25], suggesting a high hepatic extraction. Consistently, adipose tissue, a slowly perfused organ, showed no accumulation after a single dose, with an average 44-fold increase after 4 weeks of continuous exposure [53]. Compounds with high hepatic extraction, also known as flow-limited, are mainly cleared during the first pass, with a small fraction reaching systemic circulation. In the cirrhotic liver, sinusoidal capillarization and portosystemic shunt impair blood contact with hepatocytes, reducing hepatic extraction and leading to the increased bioavailability of flow-limited compounds [54]. On the contrary, compounds with low hepatic extraction are also known as enzyme-limited, and show retarded elimination with an unchanged maximum plasma concentration and bioavailability [21]. Limonene breath profile mimics the PK of flow-limited drugs, suggesting high hepatic extraction. This observation is consistent with the organ distribution observed in mice by oral and inhalation intake, with the majority of limonene found in the liver within 30 min post administration [25]. Consistently, in this study, we observed a higher limonene bioavailability in subjects with signs of portal hypertension, indicating that a dynamic limonene breath test could be used to monitor the efficacy of treatments for this complication. Interestingly, hepatic microcirculation deteriorates already at pre-cirrhotic stages [55], suggesting a potential for earlier-stage liver disease detection.

The detection of cirrhosis and earlier-stage liver diseases achieved an excellent performance in large cohort studies reporting cross-validated algorithms that combine multiple non-invasive diagnostic methods [11,12]. However, many of these parameters are generated with tests that are available in secondary and tertiary care. The sub-optimal performance of methods used in primary care leads to undetected cases that remain unreferred to follow-up [10]. This short-circuit in the diagnostic pathway explains the reports of up to 75% of cirrhosis cases being diagnosed at advanced stages with overt decompensation [1,3,4]. Strikingly, the SEAL screening program revealed that 50% of subjects with suspected cirrhosis did not attend their appointment with liver specialists and neglected further diagnostic workups [4].

Compared to the current diagnostic availability for cirrhosis, dynamic breath analysis offers several advantages. It does not require extensive training, because it consists of compound(s) ingestion followed by timely breath collection; additionally, compound(s) administration boosts breath levels, preventing the need for highly sensitive detection methods or long breath collections. For example, by using selected ion flow tube mass spectrometry (SIFT-MS), we measured the breath levels of limonene at a few parts per billion (PPB) before administration, and up to 5–10 parts per million (PPM) (>1000 fold) after administration from one 15 s exhalation and obtained real-time results (unpublished data). The development of miniaturized sensors [56,57] in portable devices, facilitates the implementation of this approach in primary care or at-home self-testing, with the consequent benefits to patients’ quality of life, especially when monitoring disease progression/regression after therapeutic interventions.

Baseline limonene breath measurement represents a static test. Although this is more practical compared to a dynamic approach, dynamic limonene measurement boosts classification performance, especially sensitivity. The misclassified subjects with cirrhosis allocated by our model are all CP class A (score = 5) with a MEDL score ≤ 8. They showed missing data points, a breath profile with a low spike after administration, or a fast decay after the peak. These limonene exhalation kinetics may be explained by technical problems, slow adsorption, faster CYPs-metabolizing variants, and/or lower alterations in hepatic perfusion. However, test performance can be further improved by using formulations with higher bioavailability, and combining multiple compounds metabolized by the liver that are detectable in breath [18].

One study strength is the extensive characterization of the participants, especially the confirmation of liver condition by ultrasound. This approach led us to identify one subject with pre-symptomatic liver disease who was enrolled in the control group and described as a case report. Strikingly, this subject was correctly allocated by our classification model, providing additional evidence of its real-life utility in diagnostics. Similarly, a participant who showed regression of morphological changes in cirrhosis, was classified as healthy, providing insight into treatment efficacy applications.

One study limitation, stemming from the necessity of maintaining a simpler study design, is the lack of blood collection at the same timepoints when breath was collected. Determining limonene blood concentration allows for an estimation of the exact blood/air ratio. However, compounds’ exhalation kinetics in relation to blood concentrations have been determined either experimentally [58] or by modelling [14], and showed consistent ratios. This evidence, together with available limonene PK information from rodents [24] and humans [52], led us to conclude that this limitation does not affect the strength of data interpretation.

The risk of selection bias was partially mitigated by enrolling subjects with earlier-stage cirrhosis and morphometric matching controls. The majority of the cirrhosis cohort are Child–Pugh class A and in the lower range of the MELD score spectrum. Since the test showed a correlation with MELD, a higher diagnostic performance is expected for advanced stages.

While we obtained a significantly younger control population, a linear mixed-effect model test indicated that age has no effect on exhalation kinetics. Nevertheless, additional studies with a screening set-up in at-risk populations are essential to validate primary care performance.

## 5. Conclusions

This study demonstrates, for the first time, the potential of breath analysis with a dynamic set-up to develop a non-invasive diagnostic test that can be implemented in primary care to enhance cirrhosis detection and prognosis.

## Figures and Tables

**Table 1:** Subjects’ characteristics.

	Control	Cirrhosis	*p*-Values
Number of patients	29	29	
Age median [IQR] Years	43 [38–57]	59 [54–67]	<0.001
Sex, *n* (%)			
Male	11 (38%)	9 (31%)	
Female	18 (62%)	20 (69%)	
Height median [IQR] cm	163 [158–170]	160 [156–170]	0.32
Weight median [IQR] kg	75 [64–84]	78 [68–85]	0.50
BMI median [IQR]	26.8 [23.9–31.2]	27.7 [26.0–32.8]	0.23
Waist circumference median [IQR] (cm)	90 [81.5–104]	106 [97–113]	0.036
Child–Pugh class	-		
A		23 (78%)	
B		5 (16%)	
N/A		2 (6%)	
MELD median [IQR]	-	10 [7.2–12.8]	
FIB4 median [IQR]	1.4 [0.8–3.1]	2.3 [1.9–4]	*p* < 0.001
APRI median [IQR]	0.2 [0.2–0.3]	0.6 [0.4–0.9]	*p* < 0.001
Platelets median [IQR] ×10^9^/L	240 [216–294]	147 [107–209]	*p* < 0.001
Total bilirubin median [IQR] (µmol/L)	8.2 [6.5–12.9]	14.3 [8.2–18.4]	*p* < 0.001
Serum albumin median [IQR] (g/L)	45 [44–45]	40 [37–44.5]	*p* < 0.001
INR median [IQR]	1 [1–1.06]	1.2 [1.0–1.4]	*p* < 0.001
ALT median [IQR] (IU/L)	18 [15–26]	28.5 [18.7–38]	*p* < 0.001
AST median [IQR] (IU/L)	21 [18.5–24]	35 [27.2–45.7]	*p* < 0.001
GGT median [IQR] (IU/L)	24 [16–32]	76 [59.2–108.7]	*p* < 0.001
ALP median [IQR] (IU/L)	85 [71.5–101.5]	117 [94.5–153]	*p* < 0.001
Creatinine median [IQR] (mg/dL)	0.78 [0.68–1.93]	0.74 [0.64–0.87]	0.31
Sodium median [IQR] (mM)	141 [138.7–142.5]	142 [139.5–142.5]	0.65

IQR: interquartile range; BMI: body mass index; MELD: model for end-stage liver disease; FIB4: Fibrosis-4; APRI: AST to Platelet Ratio Index; INR: international normalised ratio; ALT: alanine transaminase; AST: aspartate aminotransferase; GGT: gamma–glutamyl transferase; ALP: Alkaline phosphatase; N/A: not available.

**Table 2:** Limonene exhalation kinetic parameters.

Parameter	Control	Cirrhosis	*p*-Value
Cmax (ng) median [IQR]	595 [361–903]	2077 [1051–4260]	<0.001
Log10 C0 (ng) median [IQR]	6.9 [6.69–7.29]	8.4 [7.9–8.9]	<0.001
Tmax, n (%)			
20 min	18 (62.1%)	13 (44.8%)	
40 min	11 (37.9%)	12 (41.4%)	
60 min	0	2 (6.9%)	
90 min	0	1 (3.4%)	
120 min	0	1 (3.4%)	
AUC (0–90 min) ng × min/400 mL median [IQR]	27,107 [17,605–34,946]	121,437 [57,921–202,733]	<0.001
Slope	−0.027 [−0.031–−0.023]	−0.025 [−0.027–0.019]	0.072

IQR: Interquartile range; AUC: area under the curve.

**Table 3:** Summary of limonene diagnostic performances at different timepoints.

Timepoint (min)	AUROC	Sensitivity/Specificity	+/− Predictive Values (%)	+/− Likelihood Ratios
0	0.83 ± 0.12	0.66 ± 0.09/ 0.83 ± 0.07	79.17/70.59	3.8/0.42
20	0.92 ± 0.07	0.82 ± 0.095/ 0.79 ± 0.08	79.88/81.62	3.97/0.23
40	0.94 ± 0.06	0.79 ± 0.08/ 0.9 ± 0.06	88.37/80.71	7.6/0.24
60	0.91 ± 0.07	0.83 ± 0.07/ 0.9 ± 0.06	88.89/83.87	8.0/0.19
90	0.91 ± 0.07	0.76 ± 0.08/ 0.86 ± 0.06	84.62/78.12	5.5/0.28
120	0.93 ± 0.06	0.83 ± 0.07/ 0.86 ± 0.06	85.71/83.33	6.0/0.2

## Data Availability

The data presented in this study are available on request from the corresponding author. The data are not publicly available due to privacy or ethical reasons.

## Supplementary Materials

The following supporting information can be downloaded at: https://www.mdpi.com/article/10.3390/biomedicines11112957/s1. Supplementary File S1: Breath Biopsy Collection; Supplementary File S2: Limonene measurements; Supplementary Figure S1: Breath profile of the 3 subjects showing an R2 < 0.8 in Figure 1A; Supplementary Figure S2: Breath profile of misclassified subjects. Supplementary Figure S3: Correlation matrix visualizing the Spearman correlation between indicated variables. References [1,59] are cited in the supplementary.
