# Supplementary material for: Exogenous Volatile Organic Compound (EVOC®) Breath Testing Maximizes Classification Performance for Subjects with Cirrhosis and Reveals Signs of Portal Hypertension
Source: Biomedicines. 2023 Nov 1;11(11):2957. doi: 10.3390/biomedicines11112957 (PMC10669625; doi:10.3390/biomedicines11112957)
Supplement: Supplementary file 1 [file biomedicines-11-02957-s001.zip › biomedicines-2669917-supplementary.pdf]

S1. Breath Biopsy Collection

Breath collection was performed in a single room at the Functional Studies Laboratory, Clínica Alemana de Santiago, or endoscopy unit, Hospital Padre Hurtado (Santiago, Chile), for all subjects involved in the study. Breath samples were collected by adsorption onto C2-CXXX-5149 Bio-monitoring-inert-coated tubes with Tenax TA/carbograph 5TD adsorbent material (Markes International, Llantrisant, UK) through the ReCIVA® Breath Sampler (Owlstone Medical, Cambridge, UK) [1]. Before sampling, the tubes were conditioned in a TC-20 (Markes International) by a N_2 flow at 20 psi and 320 °C for 2 h. Before sampling, the system was calibrated and adjusted based on the breathing pattern of the subject. Approximately 500 mL of breath was sampled in each tube at 225 mL/min. Ambient contamination was minimized using the CASPER® Portable Air Supply (Owlstone Medical, Cambridge, UK) [59]. Additional ambient blanks were collected for each session, inside the room where breath collection was performed, to track any environment contamination. Ambient collection was performed using a procedure similar to that used for breath sampling, except that room air was directed into the sorbent tubes bypassing the CASPER air supply. The tubes were purged with a TD-100 (Markes International Ltd. Llantrisant, UK) and stored at a temperature of 4–8 °C for no more than four weeks before analysis.

S2. Limonene measurements

Samples were analyzed using an Orbitrap Exploris 60, a Trace 1310 Gas chromatograph (Thermo Fisher Scientific Inc. Waltham, MA, USA) coupled with TD 100xr thermal desorption system (Markes International Ltd., Llantrisant, UK). Samples were first pre-purged for 1 min at 50 mL/min, then desorbed at 250 °C for 10 min at the same flow and focused onto a cold trap (U-T12ME-2S, Material Emissions, C4-C32 Markes International Ltd. Llantrisant, UK) at 10 °C. Analytes were then desorbed at 250 °C for 4 min into the GC column (Stabilwax-DA 30 m × 0.25 mm × 0.25 µm, Restek Corporation, Bellefonte, PA, USA) at 2 mL/min helium flow with a on the injection of 100:1. A split ratio of 1:1 was used for baseline. The GC temperature program was as follows: 40 °C held for 4 min, increasing to 250 °C at a rate of 15 °C/min, and held for 5 min. The ion mass detection was performed using electron ionization (EI) with a mass scan range from *m/z* 30 to *m/z* 450 with resolution of 60,000. EI voltage was set at 70 eV. The ion source and transfer line temperatures were 270 and 250 °C, respectively. Limonene identification and peak integration were performed with the Chromeleon Chromatography Data System (Thermo Fisher Scientific) by injecting a limonene standard into same type of sorbent tube and analysing them together with the breath samples (62118; Sigma Aldrich, Gillingham, UK). Limonene peak was identified using accurate m/z calculation of the main MS fragments: 93.06988, 67.05423, 79.05423, 91.05423, 121.1012. Identification criteria were: mass error < 5ppm, ion ratio < 20% based on analytical standard comparison, and retention time shift < 0.1 min. The m/z of 67.05423 was used as the

quantitative mass component fragment. Areas in counts*min were used to extrapolate limonene amount in ng/500 ml of breath by comparison with a limonene standard curve (15–2000 ng on-tube).

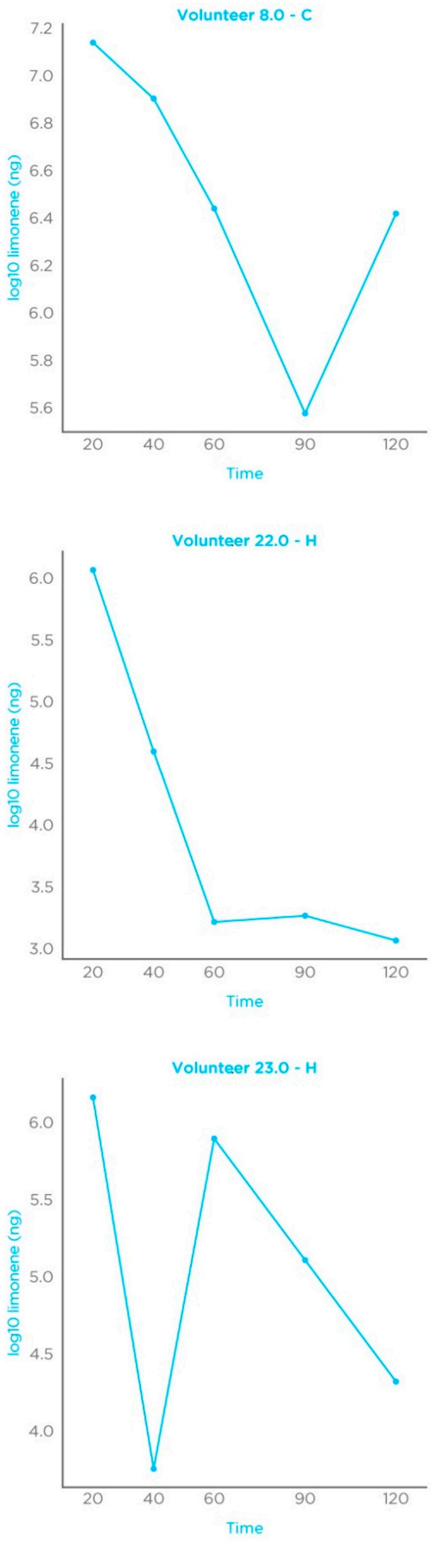

Supplementary Figure S1. Breath profile of the 3 subjects showing an R2 < 0.8 in Fig 1A.

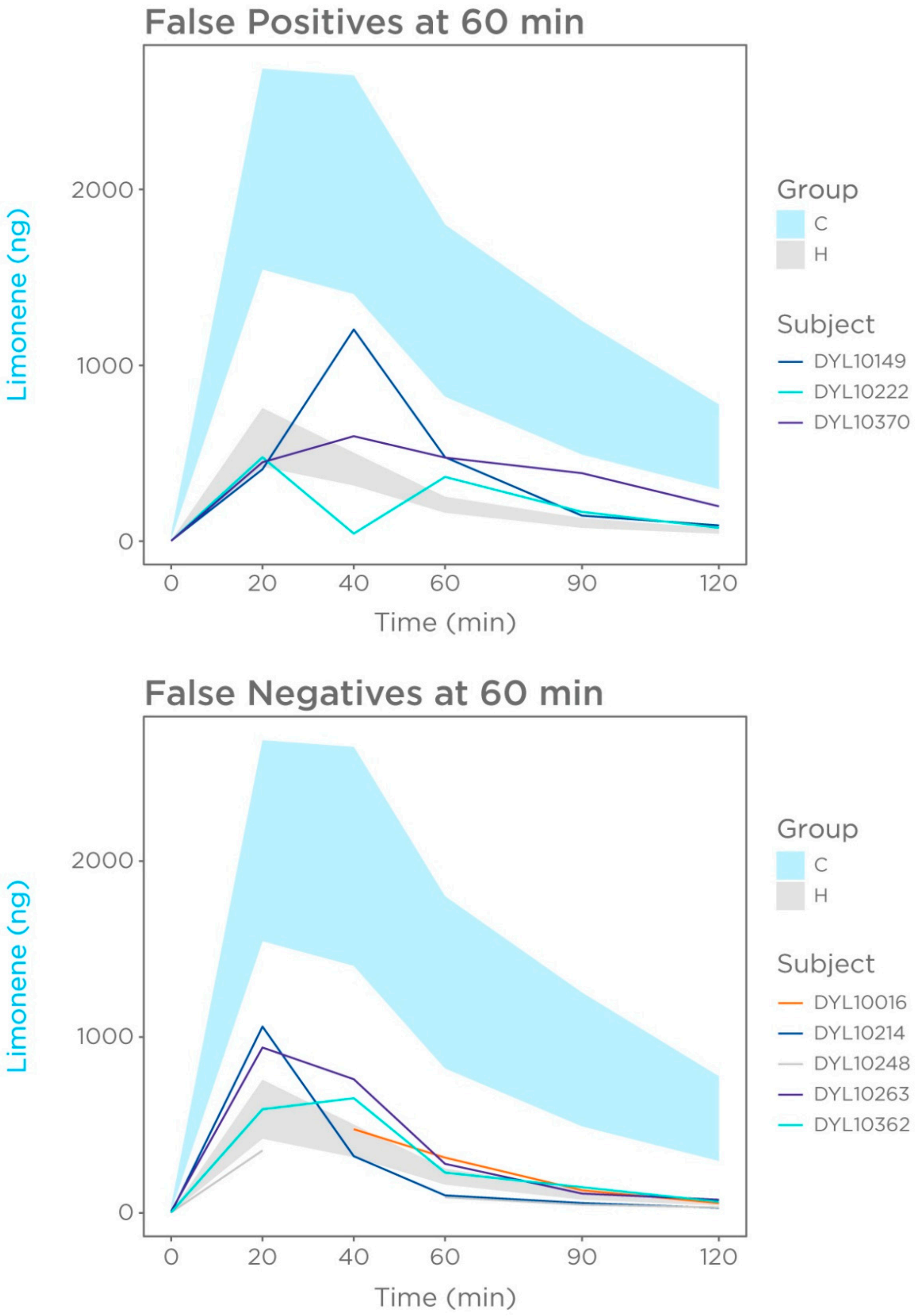

Supplementary Figure S2. Breath profile of misclassified subjects. (A) false positives, (B) false negatives. Shaded areas represent the 95% confidence interval for the cirrhosis group (blue) and control (grey) for comparison. Each line represents a subject.

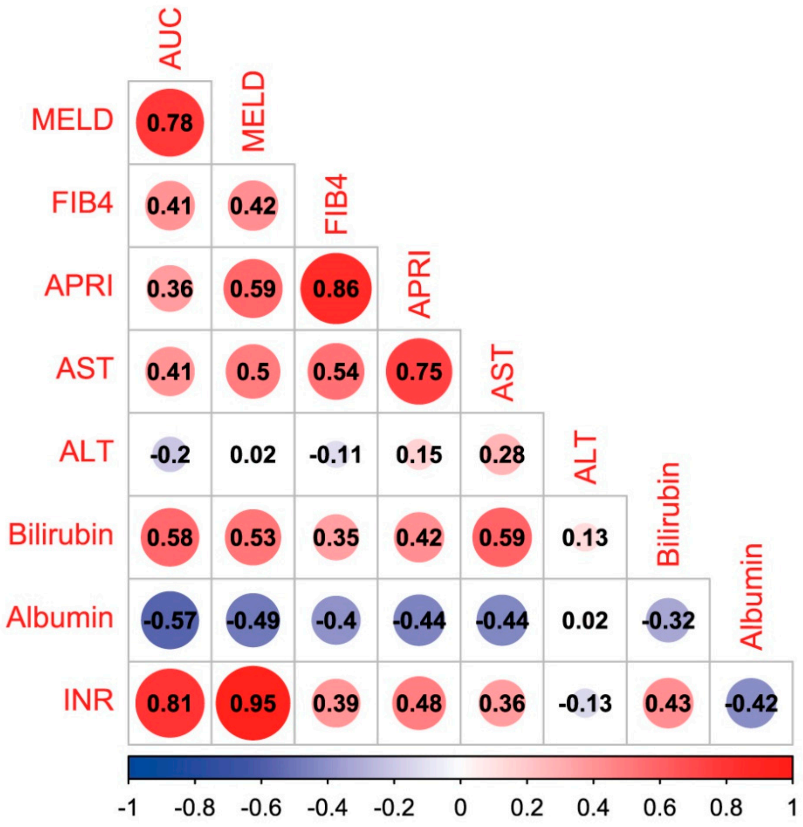

Supplementary Figure S3. Correlation matrix visualizing the Spearman correlation between indicated variables. Intensity of the red and blue colors indicate strength of respectively positive and negative correlations, while circle size represents the significance test between the variables.
